# Diagnosis and treatment of primary hyperparathyroidism with pathological fracture of the limbs: A retrospective observational study

**DOI:** 10.1097/MD.0000000000029966

**Published:** 2022-08-19

**Authors:** Huijiang Liu, Kai Luo, Shijie Liao, Haijun Tang, Jianming Mo, Tianyu Xie, Chong Li, Boxiang Li, Yun Liu, Xinli Zhan

**Affiliations:** a Department of Orthopedics, The First Affiliated Hospital of Guangxi Medical University, Nanning, Guangxi, China; b Department of Orthopedics, The First People’s Hospital of Nanning, Nanning, China; c School of Basic Medicine, Guangxi Medical University, Nanning, Guangxi, China; d Department of Orthopedics, The Minzu Affiliated Hospital of Guangxi Medical University, Nanning, Guangxi, China.

**Keywords:** primary hyperparathyroidism, pathological fracture, diagnosis, treatment

## Abstract

Primary hyperparathyroidism (PHPT) with pathological fracture is rare, and the early symptoms of PHPT lack specificity, leading to misdiagnosis. Therefore, this study aimed to summarize the clinical characteristics and treatment of PHPT patients with pathological fractures and to improve the attention of orthopedic clinicians to PHPT.

It is a retrospective study, 2226 patients with hyperparathyroidism in our hospital from 2009 to 2019 were screened, excluding secondary hyperparathyroidism and patients without limb fracture, and the remaining 20 patients with PHPT accompanied by pathological fractures were finally analyzed. Parathyroid hormone (PTH) and calcium levels were compared on the first postoperative day, and the prognosis of the patients was assessed by bone mineral density and Visual Analogue Scale scores at 3 and 12 months postoperatively.

The early symptoms of PHPT patients in this study included urinary calculi (80%), bone pain (30%), and digestive tract symptoms (25%). Fourteen (70%) cases were misdiagnosed at the initial diagnosis. After parathyroidectomy, the blood calcium and PTH levels decreased significantly in all patients (*P* < .05). For the treatment of fracture, 9 of the patients underwent surgical treatment of the fracture, while the remaining patients received splint external fixation. The follow-up time was 4.60 ± 0.62 years (1–10 years). All patients recovered well from the fracture, the symptoms of systemic bone pain were markedly improved, and bone mineral density was significantly improved after surgery.

Orthopedic surgeons need to avoid misdiagnosis and pay attention to the early symptoms in PHPT patients with pathological fracture, and better therapeutic effects can be obtained by combining parathyroidectomy with fractures fixation.

## 1. Introduction

Primary hyperparathyroidism (PHPT) is a rare endocrine disorder. PHPT is mainly caused by excessive synthesis and secretion of parathyroid hormones (PTH) by parathyroid adenomas (85%), hyperplasia (10%), or cancer (<1%),^[1,2]^ which leads to high blood calcium levels and reduced blood phosphorus levels, thus causing a series of pathological changes in the bone and kidney.^[3]^ PHPT can be categorized into the kidney, bone and kidney-bone types. The early manifestations of skeletal hyperparathyroidism include osteoporosis, diffuse, and progressive pain of the skeletal joints throughout the whole body and in load-bearing parts, especially the hip joint.^[4,5]^ In the late stage, reactive fibrous connective tissue hyperplasia leads to pseudotumor lesions due to extensive absorption of the cortex, manifested as fibrocystic osteopathy.^[6]^ Therefore, a slight external force can lead to pathological or spontaneous fractures. Parathyroidectomy is the gold standard treatment for PHPT patients, with a surgical cure rate of 95% to 98%, as it can effectively inhibit bone resorption, and reduce the incidence of complications, such as fractures.^[7–11]^

The incidence of PHPT ranges from 0.4 to 82 cases per 100,000. The incidence of pathological fractures associated with PHPT is even lower, accounting for 16.4% of patients with PHPT.^[11,12]^ Patients often seek treatment in orthopedics for pathological fractures of the limbs caused by minor trauma. Due to the lack of understanding of this disease by orthopedic surgeons, it is easily confused with common clinical osteoporosis or neoplastic pathological fractures, resulting in misdiagnoses. The misdiagnosis rate of PHPT is high in China. If patients cannot receive timely and appropriate treatment owing misdiagnosis, bone destruction will continue to deteriorate, often leading to repeated pathological fractures.

Most of the previous literatures have focused on the diagnosis and treatment of PHPT. Only a few cases of the diagnosis and treatment of pathological fractures complicated by PHPT have been reported, and there are even fewer systematic case review analyses. The diagnosis and treatment of this disease remain challenging in the clinic. Therefore, in this study, we retrospectively analyzed 20 patients diagnosed with both PHPT and pathological fracture in our hospital, summarized their diagnosis and treatment experiences, and provided references for clinicians, especially orthopedic surgeons. To our knowledge, this is the first systematic case report of this disease with a large sample size.

## 2. Materials and Methods

### 2.1. Date sources

This is a retrospective study. All patients with hyperparathyroidism who were referred to the First Affiliated Hospital of Guangxi Medical University from January 2009 to December 2019 were recruited. The inclusion criteria were as follows: (1) Primary hyperparathyroidism, as confirmed by biochemical and imaging examinations and a pathological examination; (2) Pathological fracture in the limbs; (3) Complete records of the diagnosis, treatment, follow-up, and a follow-up time of more than 1 year. Exclusion criteria were as follows: (1) Pathological fracture caused by secondary hyperparathyroidism; (2) Incomplete records on diagnosis, treatment and follow-up; (3) The follow-up period was <1 year.

**Figure 1.**
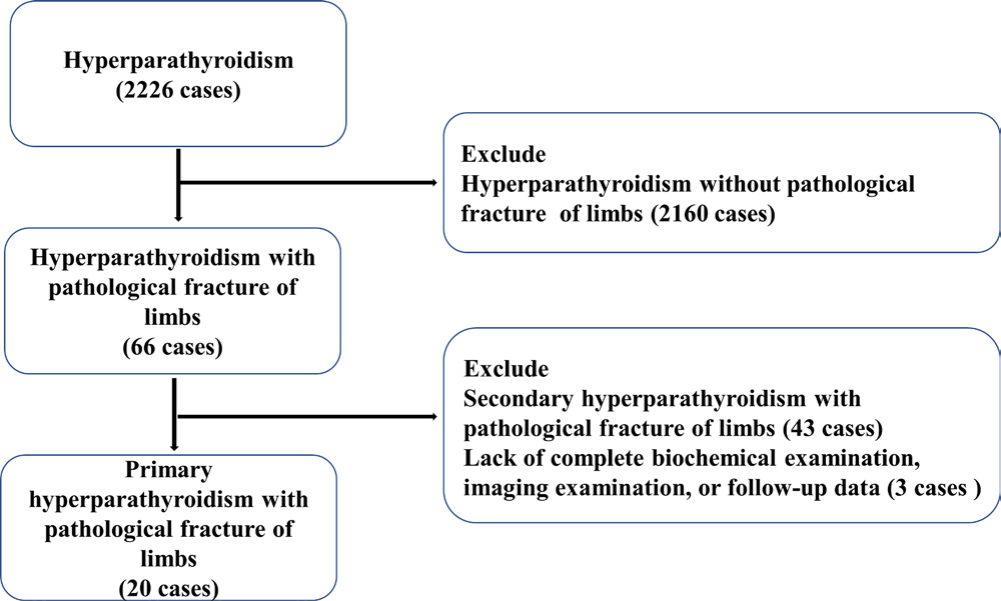
Flowchart.

A total of 2226 patients with hyperparathyroidism were included in this study, including 66 patients with pathological limb fractures. According to our inclusion and exclusion criteria, 43 patients with secondary hyperparathyroidism were excluded, 2 patients were excluded due to incomplete biochemical examination and imaging data, and 1 patient was excluded due to incomplete follow-up data. Finally, 20 patients were included in the study (Fig. 1). The study protocol was approved by the Ethics Committee of the First Affiliated Hospital of Guangxi Medical University in January 2019 (Number: 2018 (KY-E-129)). Given the retrospective nature of this study, informed consent was not required.

### 2.2. Therapeutic methods

All patients in this study underwent parathyroidectomy, which is the only effective treatment for PHPT. Hypocalcemia develops easily after parathyroidectomy. Therefore, calcium and vitamin D supplements should be improved. Surgical treatment was recommended for all patients in this study for fracture treatment. For patients with contraindications, drugs and external splint fixation can be used first, and further surgery can be performed after BMD is improved.

### 2.3. Follow-up

All patients were required to return to the outpatient clinic of our hospital for re-examination at the 1st, 3rd, 6th and 12th months after surgery and once a year after 12 months. Follow-up included the evaluation of PTH, serum calcium, serum phosphorus, ALP and other biochemical indexes, as well as Visual Analogue Scale, and limb function scores. To better understand healing and bone mass recovery at the fracture site, radiographic and bone mineral density (BMD) examinations of the fracture site were performed regularly for each patient.

### 2.4. Statistical analysis

The data were statistically analyzed using the statistical software SPSS 24.0. Measurement data are expressed as mean ± standard deviation. Intragroup comparisons were performed using paired t-tests, and comparisons between groups were performed independent sample t-tests. The count data were expressed by the number of cases and percentages, and comparisons between groups were performed by ×2 test. *P* < .05 was considered statistically significant. *P* < .01 was considered obviously statistically significant.

## 3. Results

### 3.1. Clinical characteristics

Twenty patients were included in this study (Fig. 1). There were 5 males aged 19 to 29 and 15 females aged 30 to 64. According to the pathological classification of PHPT, there were 11 cases of parathyroid adenoma, 9 cases of parathyroid hyperplasia. The PHPT patients in this study had mainly femoral fractures, and there were 15 cases of femoral fracture, 1 case of a hidden fracture of the middle and upper fibula, 2 cases of a surgical neck fracture of the humerus, and 2 cases of a distal radius fracture. Among the complications of PHPT in this study, 16 patients had urinary calculi, 6 patients had systemic bone pain or joint pain, and 5 patients had gastrointestinal symptoms. Other clinical signs included fatigue, dry mouth, and polydipsia (Table 1). In terms of misdiagnoses, 14 (70%) patients were initially misdiagnosed with pathological fractures caused by osteoporosis, giant cell tumors, etc, with an average delay in diagnosis of 11.80 ± 2.92 months (1–36 months). Because of misdiagnoses, the fracture site was only treated with reduction and internal fixation instead of parathyroid gland resection, leading to 8 patients repeatedly suffering pathological fractures at the same site or other sites due to minor trauma. The average cumulative number of fractures was 1.8 ± 0.22 (1–5 times).

**Table 1:** The clinical characteristics of primary hyperparathyroidism with pathological fracture of the limbs.

Case	Sex	Ages (yr)	The site of fractures	Fatigue	Pain in the bones or joints of the whole body	Dry mouth, excessive drinking, and urination	Complication	Pathological type	Treatment of fracture
1	Male	19	Bilateral femur	Yes	Yes	No		Parathyroid adenoma	Plate internal fixation
2	Female	39	Middle of right femur	No	Yes	No	Acute pancreatitis, kidney stone, high calcium crisis	Parathyroid adenoma	Plate internal fixation
3	Male	29	Surgical neck of left humerus	No	No	No		Parathyroid hyperplasia	Splint external fixation
4	Female	56	Right intertrochanteric fracture	Yes	No	Yes	Kidney stone	Parathyroid adenoma	Splint external fixation
5	Female	41	Right femur	No	Yes	No	Kidney stone	Parathyroid adenoma	Plate internal fixation
6	Female	48	Left fibula	No	No	No	Kidney stone	Parathyroid hyperplasia	Splint external fixation
7	Female	64	Right radius	Yes	Yes	No	Kidney stone	Parathyroid hyperplasia	Plate internal fixation
8	Female	30	Bilateral femur	No	No	No	Kidney stone, pseudocyst of pancreas	Parathyroid hyperplasia	Splint external fixation
9	Female	35	Left femur and fourth metacarpal	No	Yes	No	Kidney stone	Parathyroid adenoma	Splint external fixation
10	Male	24	Bilateral femur	Yes	No	Yes		Parathyroid adenoma	Splint external fixation
11	Female	38	Bilateral femur	Yes	No	Yes	Kidney stone, pseudocyst of pancreas	Parathyroid hyperplasia	Plate internal fixation
12	Male	23	Right femur	Yes	Yes	No		Parathyroid adenoma	Splint external fixation
13	Male	25	Left radius	Yes	No	No	Kidney stone	Parathyroid adenoma	Plate internal fixation
14	Female	55	Left femur	No	No	No	Kidney stone	Parathyroid hyperplasia	Splint external fixation
15	Female	40	Right femur	No	No	Yes	Acute pancreatitis, kidney stone, high calcium crisis	Parathyroid adenoma	Splint external fixation
16	Female	71	Surgical neck of right humerus	No	No	No	Kidney stone, pseudocyst of pancreas	Parathyroid adenoma	Plate internal fixation
17	Female	50	Left intertrochanteric fracture	No	No	No	Kidney stone	Parathyroid hyperplasia	Plate internal fixation
18	Female	60	Right femur	No	No	No	Kidney stone	Parathyroid adenoma	Plate internal fixation
19	Female	30	Left femur	No	No	No	Kidney stone	Parathyroid hyperplasia	Splint external fixation
20	Female	32	Right femur and fourth metacarpal	Yes	No	No	Kidney stone	Parathyroid hyperplasia	Splint external fixation

According to the laboratory examinations, the preoperative PTH level in 20 patients (100%) was far beyond the normal value. The preoperative blood calcium level of 18 patients (90%) exceeded the normal value, and that of 2 patients was slightly lower than the normal value, but all cases were lower than the normal value of blood phosphorus. Alkaline phosphatase (ALP) is secreted by osteoblasts. Therefore, its value reflects the extent of the bone loss. In our study, ALP values were higher than normal in 19 patients (95%) and normal in 1 patient.

### 3.2. Imaging examination

All PHPT patients were diagnosed by neck B-ultrasound or radionuclide and verified by pathology. In terms of imaging of the skeletal system, all 20 patients underwent whole-body X-ray examination, which showed that the BMD of patients had decreased in different degrees. Seventeen patients had osteoporosis. The X-rays showed subperiosteal bone resorption, thinning of the bone cortex and trabecular bone, and blurring of the edges. In this study, 18 patients had cystic fibrous osteitis on the X-rays, including 5 patients with single-site cystic fibrous changes and 13 patients with multisite cystic fibrous changes. Regarding the common site of cystic fibrous osteitis, 12 cases occurred in the femur, 11 occurred in the pelvis, 4 occurred in the scapula, and the remaining cases occurred in the spine, ribs, patella, humerus, and fibula. To differentiate the diagnosis from those of other diseases, other imaging examinations of the skeletal system were performed: 9 patients underwent plain CT or plain CT + enhancement scans, 4 underwent plain MRI scans, and 2 underwent whole-body bone ECT scans.

### 3.3. Clinical effect and follow-up

**Figure 2.**
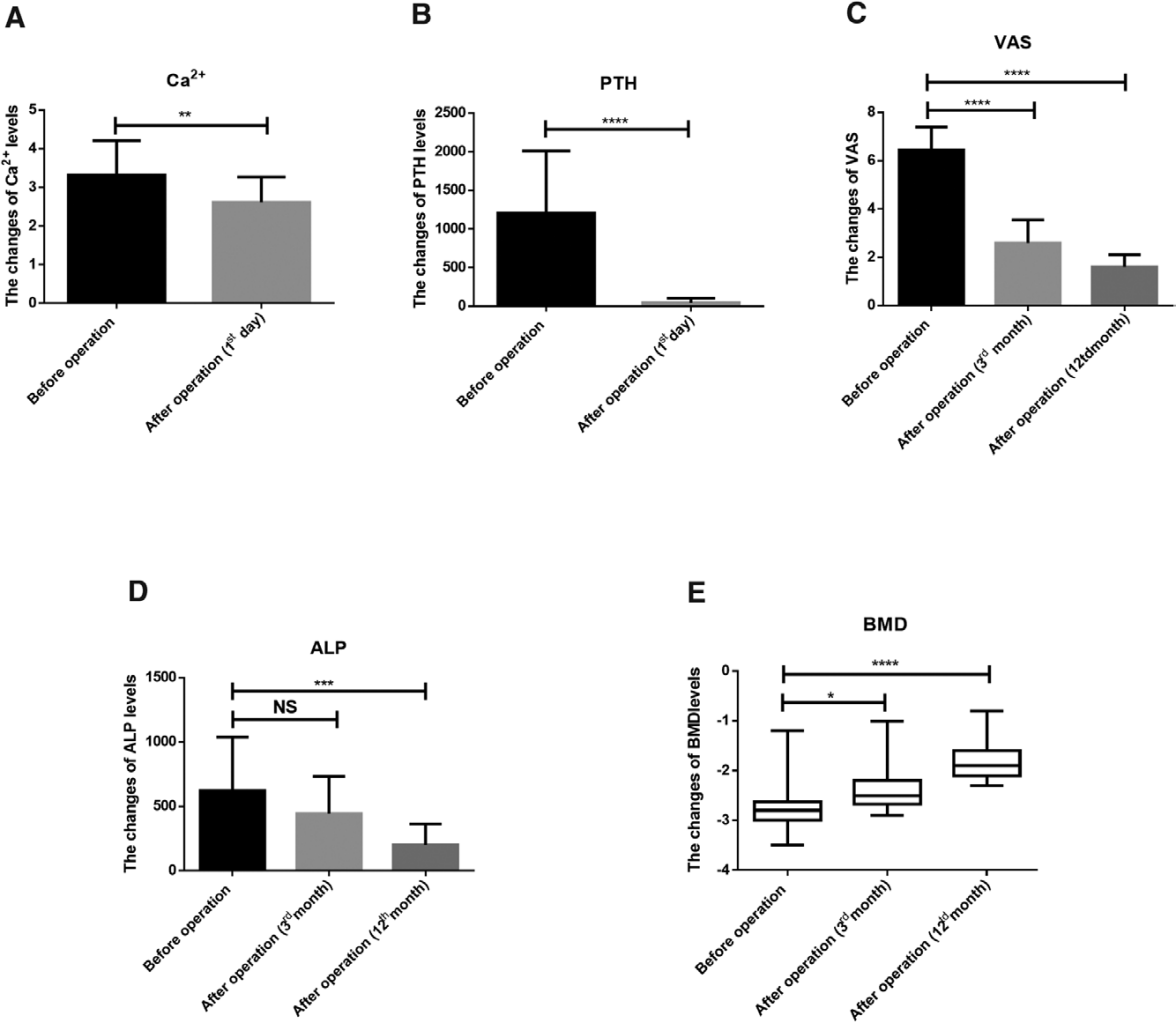
The results of biochemical examination and follow-up data. (A) The results of PTH levels before parathyroidectomy and on first day after parathyroidectomy. (B) The results of Ca^2 +^ levels before parathyroidectomy and on first day after parathyroidectomy. (C) The changes of VAS before parathyroidectomy, at 3rd month and 12th month after parathyroidectomy. (D) The changes of ALP before parathyroidectomy, at 3rd month and 12th month after parathyroidectomy. (E) The changes of BMD before parathyroidectomy, at 3rd month and 12th month after parathyroidectomy. ALP = alkaline phosphatase, BMD = bone mineral density, PTH = parathyroid hormone, VAS = Visual Analogue Score.

**Figure 3.**
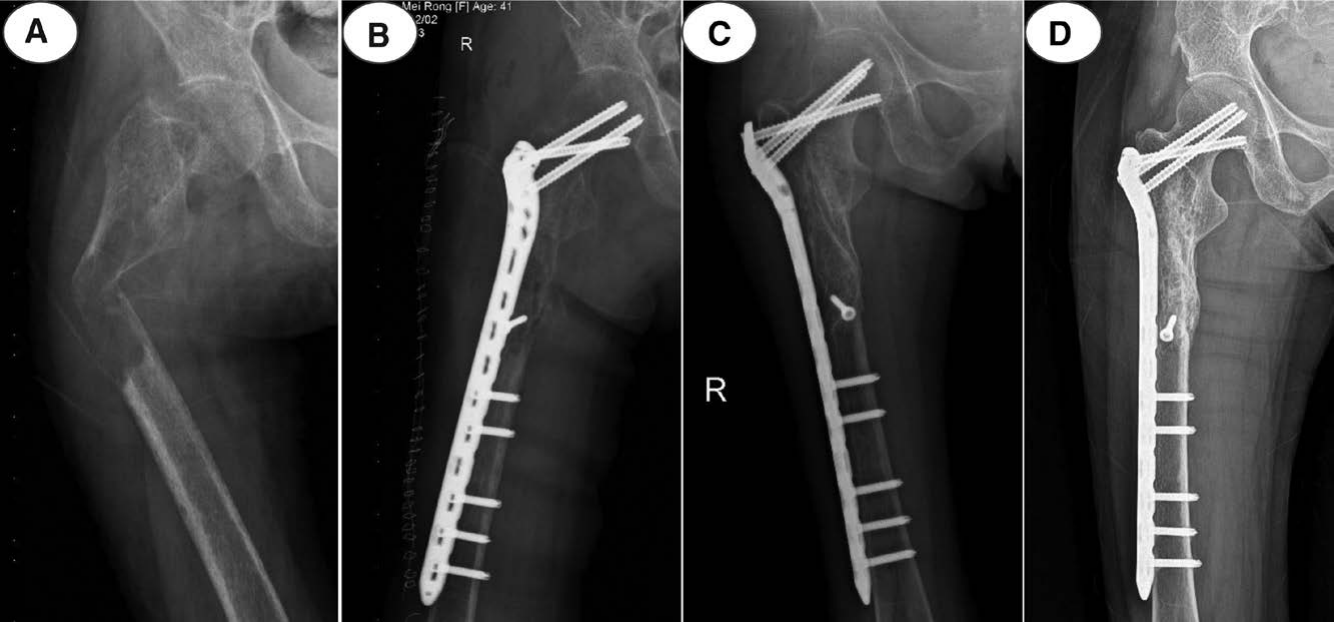
Typical case: a 41 years old female, primary hyperparathyroidism with pathological fracture of right proximal femur. (A) X-ray shows widely osteoporosis, obvious displacement of fracture site, subperiosteal bone absorption, and fibrocystic osteitis; (B) The patient was treated with bone grafting and internal fixation after parathyroidectomy; (C) The fracture line disappeared and fracture healed well 3 months after operation; (D) Bone mineral density of the patient increased significantly and osteoporosis was cured 4 years after operation.

Twenty patients underwent neck parathyroid resection after the diagnosis. For the treatment of pathological fractures of the limbs, 9 patients chose internal fixation for fracture reduction, and 11 patients chose conservative treatment (Table 1). All patients were followed up by telephone, WeChat (a Chinese social media application software) or outpatient review for 1 to 10 years, and the average follow-up time was 4.60 ± 0.62 years. The result showed that the PTH of patients after parathyroidectomy decreased from 1207 ± 179.8 ng/L before the operation to 46.39 ± 13.10 ng/L on the first day after the operation, and the difference was statistically significant (*P* < .0001) (Fig. 2A). The same decreased changes were found in the level of blood calcium (2.61 ± 0.14 mmol/ L vs 3.33 ± 0.19 mmol/ L, *P* = .0062) (Fig. 2B). ALP is an osteoblast-related index, and the results show that the ALP levels at 3th month and 12th month after surgery decreased, indicating that parathyroidectomy alleviated further aggravation of bone damage (Fig. 2C). In terms of the pain score, the Visual Analogue Scale scores before parathyroidectomy at 3th month and 12th month after parathyroidectomy were compared in this study, and the difference was statistically significant, suggesting that parathyroidectomy can relieve the bone pain caused by PHPT (Fig. 2 D). The BMD of the hip was assessed at 3th month and 12th month after parathyroidectomy, and the results showed that the T value increased from −2.76 ± 0.12 before the operation to −1.77 ± 0.10 at 12th month after the operation (*P* < .0001) (Fig. 2 E), suggesting that parathyroidectomy can further reduce the loss of bone and improve the bone density of patients, and the typical case also supports this result (Fig. 3). Statistical analysis of the clinical efficacy of the treatment for pathological fractures of the limbs was also conducted. All patients achieved bone healing regardless of whether they received conservative treatment or underwent internal fixation with a steel plate. After 1 year, 11 patients in the conservative treatment group had basically recovered the function of the affected limb, 7 patients could perform normal work, and 4 patients could perform some simple physical work. The function of the affected limb was excellent in 7 cases, good in 2 cases and poor in 2 cases, with a superior rate of 81.8%. Among the 9 patients in the surgical treatment group, 7 could perform normal work and 2 could perform simple physical work. However, 2 patients with a femoral shaft fracture did not adhere to rehabilitation exercise after surgery, resulting in knee stiffness and an inability to squat down. Five patients had excellent limb function, 2 had good function, and 2 had poor function, and the superior rate was 77.8%.

## 4. Discussion

PHPT with secondary pathological fractures is a progressive process, and the pathogenesis is closely related to the influence of elevated PTH levels on bone metabolism. Most patients with PHPT have abnormal PTH, calcium, phosphorus and ALP levels.^[13]^ In this study, all patients had increased PTH levels and decreased blood phosphorus levels before surgery. Serum calcium levels were elevated in 90% of patients before surgery, but in 2 patients with slightly lower than normal serum calcium levels, it is possible that patients with advanced PHPT may develop hypocalcemia due to massive calcium loss in the bone. In addition, all 20 patients had elevated ALP levels, which are formed and secreted by osteoblasts and can reflect the degree of bone loss. Fractures caused by PHPT are easily confused with pathological fractures caused by osteoporosis and bone tumors, and the misdiagnosis rate is extremely high.^[5]^ In this study, 14 patients (70%) were not initially diagnosed with PHPT. Therefore, clinicians should consider the diagnosis of PHPT in patients with high blood calcium and low blood phosphorus levels.

The femur is the largest tubular bone in the human body and can only be broken when hit by a large impact force. However, 15 (75%) patients had femoral fractures in this study, all of which were caused by minor trauma, indicating the severity of bone injury and a significant decrease in BMD. Therefore, orthopedic surgeons should be highly alert to pathologic fractures with minor trauma and to distinguish the causes of pathologic fractures. According to literature, the main clinical symptoms of PHPT in the early stages are bone pain (46.02%), urolithiasis (41.59%), constipation (25.66%), fatigue (18.58%), and polyuria (15.93%).^[11]^ Similarly, the 20 patients in this study also had some clinical symptoms before the pathologic fracture, 16 patients had urinary calculi, 6 patients had systemic bone pain or joint pain, and 5 patients had gastrointestinal symptoms. Other clinical signs include fatigue, dry mouth, and polydipsia. The main function of PTH is to regulate the metabolism of calcium and phosphorus. When the secretion of PTH increases, calcium in the blood increases, as does calcium in the urine, and excessive calcium can lead to calcium deposits that can form kidney stones.^[14,15]^ High serum calcium levels can also stimulate gastrin secretion and trypsinogen activation, causing gastrointestinal symptoms. Therefore, attention should be paid to the early clinical symptoms of patients, especially those with urinary calculi, and be highly alert to pathological fractures caused by hyperparathyroidism.

Neck ultrasonography and MRI can help in the diagnosis of PHPT. In this study, X-ray examination is also necessary to help evaluate the patient’s overall BMD. X-ray images of patients with PHPT showed subperiosteal bone absorption, thinning of the bone cortex and bone trabecula, and blurred edges. Kidney stones can also be found by X-ray, which can aid in further diagnosis. In addition, attention should be paid to the identification of pathological fractures caused by osteoporosis and bone tumors in elderly women. Osteoporosis often occurs in postmenopausal women clinically because estrogen can promote the growth of bone, while postmenopausal women have lower levels of estrogen secretion, thus leading to the loss of bone throughout the body and a reduction in bone density. Clinical manifestations include systemic bone and joint pain.^[16,17]^ Most PHPT patients are elderly and postmenopausal women, and the ratio of males to females is 1/2 to 3.^[2]^ The age of onset and clinical symptoms of these 2 types of patients are very similar and lack specificity, which can lead to misdiagnosis of PHPT.^[18]^ Among the 15 female patients in our study, 8 (53%) were postmenopausal. Therefore, orthopedic surgeons should be careful in distinguishing PHPT from postmenopausal osteoporosis when assessing elderly female patients with pathological fractures. Besides, giant cell tumors of the bone occur mainly in the epiphysis. X-ray findings showed thinning of the cortical bone at the lesion site, with soap-like changes.^[19,20]^

Parathyroid gland resection can significantly inhibit osteoclasts activity, significantly improve BMD, and effectively reduce the risk of pathological fractures in patients with PHPT. Therefore, scholars at home and abroad believe that once PHPT is confirmed, the parathyroid gland should be removed as soon as possible.^[8,17,21–23]^ In this study, all patients underwent parathyroid gland resection, and the PTH and blood calcium levels returned to normal levels 3 months after surgery. The ALP level decreased significantly, the BMD increased significantly compared to the preoperative level, and the ALP level and BMD were negatively correlated. One year after surgery, the imaging results showed cortical thickening and increased and thickened trabeculae. These results further demonstrate that parathyroid resection can reverse bone destruction and is the primary treatment for this disease.

For fracture treatment in PHPT patients, factors such as the age of the patient, degree of bone destruction at the fracture end, fracture site, and displacement degree should be comprehensively considered so that the physician can decide whether to indicate surgical treatment or external fixation treatment. For patients with severe osteoporosis, particularly those with middle and lower femoral fractures, surgical treatment should be performed cautiously. Conservative treatment can be performed first, followed by internal fixation when osteoporosis is improved. In this study, 9 patients were treated with surgical internal fixation of the fracture site, and 11 were treated with conservative reduction and external fixation. According to the follow-up results of this study, the superior rate of function in the surgical treatment group was not better than that in the conservative treatment group. In the surgical treatment group, 2 patients did not adhere to functional exercise after surgery, resulting in knee joint adhesion and joint dysfunction after femoral fracture. The main reason for poor outcome after surgery is femoral fracture, especially a fracture in the middle and lower segments, which increases the trauma to the knee extensor closure device and increases local blood infiltration and fibrin exudation, which can easily cause adhesion of the knee extensor device after the operation. In addition, patients should undergo functional exercise early after surgery. However, if the patient has obvious osteoporosis, the excessive strength of functional exercise can easily cause failure of internal fixation, which also limits the intensity of early functional exercise. If surgical treatment is performed, early postoperative professional functional rehabilitation and internal fixation failure should be balanced; otherwise, treatment outcomes will be affected. In addition, the degree of fracture displacement in the conservative treatment group was relatively small, and most fractures were shaft fractures. A good reduction effect can be achieved using conservative treatment. However, in patients with a high degree of displacement, postoperative malunion, and poor limb strength, there is still a risk of secondary osteoarthritis in the adjacent joints. Therefore, parathyroidectomy remains a key treatment option for this disease. Furthermore, it is necessary to integrate individual fracture end management schemes with multiple factors to achieve better treatment outcomes.

There are several limitations to our study. First, it was a retrospective observational study; it included only 20 cases, and the sample size is small. However, PHPT with pathological fracture is rare, and this study has the largest sample size to date. Second, this was a single-center study, which may have bias in the diagnosis and treatment. Therefore, a multicenter study with larger sample size is required for validation.

## 5. Conclusion

Orthopedic surgeons should pay attention to the diagnosis of PHPT in patients with pathological fractures when blood calcium and phosphorus are abnormal. X-rays and early complications are also crucial in the diagnosis of PHPT. Parathyroidectomy combined with plate fixation can achieve better results.

## Author contributions

Conceptualization: Huijiang Liu, Kai Luo, Shijie Liao

Data curation: Kai Luo, Haijun Tang

Formal analysis: Jianming Mo

Funding acquisition: Yun Liu, Shijie Liao

Investigation: Huijiang Liu, Kai Luo

Methodology: Tianyu Xie, Chong Li

Project administration: Xinli Zhan, Yun Liu

Supervision: Xinli Zhan, Yun Liu

Validation: Huijiang Liu

Visualization: Huijiang Liu, Boxiang Li

Writing—original draft: Huijiang Liu, Kai Luo

Writing—review and editing: Xinli Zhan, Yun Liu
